# Community perspectives on the built environment, community stress, and the risk of diabetes and cardiovascular diseases in Accra, Ghana

**DOI:** 10.1186/s12889-025-24106-z

**Published:** 2025-10-14

**Authors:** Mawuli Komla Kushitor, Haim Yacobi, Lydia Osetohamhen Okoibhole, Sandra Batemaa Kushitor, Publa Antwi, Irene Akwo Kretchy, Olutobi Adekunle Sanuade, Samuel Amon, Leonard Baatiema, Vida Asah-Ayeh, Hassan Haghparast-Bidgoli, Hannah Maria Jennings, Daniel Llywelyn Strachan, Carlos Salvador Grijalva-Eternod, Ann Blandford, Megan Vaughan, Edward Fottrell

**Affiliations:** 1 Department of Health Policy, Planning and Management (HPPM), Fred N. Binka School of Public Health (HPPM), University of Health and Allied Sciences (UHAS), Ho, Volta Region Ghana; 2 Development Planning Unit, Faculty of the Built Environment, University College London, London, UK; 3 Faculty of Education, University of Cambridge, Cambridge, UK; 4 Department of Community Health, Ensign Global College, Kpong, Ghana; 5 Department of Food Science and Centre for Sustainability Studies, Stellenbosch University, Stellenbosch, South Africa; 6 Department of Health Sciences, University of York, York, UK; 7 Department of Pharmacy Practice and Clinical Pharmacy, School of Pharmacy, University of Ghana, Legon, Ghana; 8 Department of Population Health Sciences, Division of Health System Innovation and Research, Spencer Fox Eccles School of Medicine, University of Utah, Salt Lake City, USA; 9 Department of Health Policy, Planning and Management, School of Public Health, University of Ghana, Legon, Ghana; 10 Centre for Tropical Medicine and Global Health Research, Nuffield Department of Medicine, University of Oxford, Oxford, UK; 11 University of Ghana, Legon, Ghana; 12 Institute for Global Health, University College London, London, UK; 13 Hull York Medical School, Heslington, UK; 14 The Nossal Institute for Global Health, University of Melbourne, Melbourne, Australia; 15 Department of Population Health, London School of Hygiene and Tropical Medicine, London, UK; 16 Department of Computer Science, University College London, London, UK; 17 Institute of Advanced Studies, University College London, London, UK

**Keywords:** Cognitive map, Community perspectives, Built environment, Stress, Cardiovascular diseases, Diabetes

## Abstract

**Background:**

The growing burden of diabetes and other non-communicable diseases in Africa demands greater understanding of the contextual drivers of risk factors, including the built environment. Cognitive Mapping (CM) is a participatory research approach that allows community members to visualise their environmental context through drawing. The maps express in visual form the situated knowledge of the environment from local perceptions of daily experiences. This study combines Geographic Information System (GIS) techniques and qualitative research methods to explore community perspectives on the environmental risk factors of diabetes and other cardiovascular diseases (CVDs) in a poor urban community in Accra, Ghana.

**Methods:**

Five Cognitive Map Focus Group Discussions (CM-FGDs) and four regular Focus Group Discussions (FGDs) were conducted with a total of 43 participants in Ga Mashie (Accra) in November and December 2022. Participants (25 women and 18 men) had lived in the study communities for over ten years. Community members were given paper and a pencil for the CM sessions to draw their environment. GIS maps supplemented the community drawings. We adopted geo-ethnography, a technique that combines GIS and qualitative analytical methods. The GIS was used to recreate aspects of the physical environment discussed by study participants. The FGDs were analysed thematically.

**Results:**

Participants recognised the physical and social attributes of their daily environment and how these attributes influence the risk of CVDs. Excessive heat and hazardous noise from overcrowded spaces emerged as key health risks. The social environment was equally important – participants often linked the high concentration of bars, spaces for social interaction and several social engagements at weekends to excessive consumption of alcohol and unhealthy food. Community members reported that social behaviour and diet associated with their environments were gradually deteriorating, and these accounted for observed changes in patterns of diabetes and related CVDs. Specifically, community members attributed the causes of hypertension and heart disease to hazardous noise and psychological distress associated with the built environment. In contrast, diabetes was generally attributed to the social environment.

**Conclusion:**

Cognitive maps allowed community members to participate in research and link the risk of diabetes and CVDs to their changing environment. Built environment interventions should empower communities to make large-scale behavioural modifications to improve the prevention and control of diabetes and CVDs within their community.

**Supplementary Information:**

The online version contains supplementary material available at 10.1186/s12889-025-24106-z.

## Background

Environmental factors play a significant role in determining risks for the occurrence and management of diabetes and other related metabolic and cardiovascular diseases (CVDs) [1–3]. These risks – including insufficient physical activity, poor diet, excessive alcohol consumption, smoking, and poor quality and quantity of sleep [4–7] – are influenced substantially by environmental characteristics [3, 8, 9]. However, for all the mediating influence of the environment, most diabetes and CVD research in Africa emphasises individual-level behavioural factors, such as motivation and agency, as determinants of modifiable risk factors [10, 11], neglecting the environment. For example, environmental noise [10] can interfere with sleep and complicate the management and risk of diabetes in urban areas [11]. Yet, insufficient consideration has been given to broader environmental determinants, such as the food environment and green spaces for physical activity. While individual agency matters, the environment is an active entity that continually interacts with people and influences their choices [9, 12]. Proponents of the built environment argue that the abundance of unhealthy foods influences individual behaviours. Sufficient research [13–18] shows that in urban areas with a high concentration of alcohol and processed foods, there is increased consumption of these products, leading to a higher burden of diabetes and CVDs [18]. In many African countries, the burden of these conditions is typically much higher in urban areas, especially in informal settlements [19–21].

Further, although the environment (including infrastructure, public spaces, housing and community spatial organisation) is an essential determinant of health behaviours, there is contradictory evidence on how the environment influences behaviour. For example, residential arrangement and local area connectivity have been reported in some studies to influence physical activity [9], but not in others [22]. Systematic reviews often attribute these contradictions to measurement differences [23]. Most of these measurements focus on the physical attributes of the built environment with limited emphasis on community involvement, engagement and perspective – a void this article aims to fill.

While the literature on Ghana recognises the influence of the built environment, the research has primarily focused on government intervention [24], such as developing and implementing policies to improve health-promoting behaviours at the structural level. Other studies highlight the gender differentials in environmental impacts and quantitative self-assessment of NCD risks [8, 25]. Existing research mostly lacks information on how the local community perceives their environment regarding health risks. New evidence, however, shows that observing the environment from the community’s perspective can lead to better-designed and more effective interventions [26]. Indeed, a growing body of urban health literature emphasises the perspectives of community members who are affected by the space they live in [23, 26]. Some scientists have suggested that any intervention proposed for the built environment should be based on the perspectives of the local community [27].

Cognitive maps (CMs) are powerful tools for visualising and voicing a community’s perception of the built environment. They illustrate local knowledge of the environment informed by history, social interactions and lived experience. As such, CMs allow researchers to measure environmental consciousness, which is often overlooked, and they document the perspectives of the environment from those who live in it and are directly affected. They provide an authentic and relevant perspective to advance intervention approaches. CMs are also participatory, allowing community members to contribute to the scientific process, and they emphasise the importance of place in health, a concept that is critical to public health [28].

This study explores local community members’ conceptualisations of their built environment and the influence of this environment on risk of diabetes and CVDs. The built environment refers to the physical and built infrastructure where people live, learn, work, socialise and travel [29].

## Methods

### Study design

This study employed an exploratory sequential mixed methods technique consisting of mixed qualitative Focus Group Discussions (FGDs) and Geographic Information Systems (GIS) mapping. The FGDs were conducted first, followed by the GIS mapping. The qualitative component of the study comprised Cognitive Mapping Focus Group Discussions (CM-FGDs) and regular FGDs. The CM-FGDs focused on the built environment and its association with diabetes and other CVDs. Subsequently, FGDs were held with food traders within the community. Finally, GIS mapping was used to recreate aspects of the environment discussed in the qualitative research, which provided spatial context to the narratives. Geo-ethnography harnesses the power of GIS to create maps and provide context to qualitative interviews [30].

CM-FGDs develop a local perspective of the environment, including the geographic extent of the community and what those boundaries mean to the community. The local construction of the environment is often based on the community’s authentic historical, social and cultural experiences.

CM is an open-ended and inclusive qualitative strategy because it allows everyone to express themselves without being limited by language, education or social barriers. Providing blank paper for individuals to express themselves enables people to creatively construct their environment without being influenced much by the researcher. Participants further explained their drawings in FGDs. Kevin Lynch, an American Architect and urban planner, pioneered this method. According to Lynch, people use five key elements to construct their perspectives of the environment [31]: paths, edges, districts, nodes and landmarks. Paths include walkways, streets, alleys and corridors. Edges refer to boundaries. Districts are areas with specific features or identities. Nodes are junctions and particular locations within the community. Landmarks refer to major known physical features in the community. These five key elements were deployed in the CMs.

This methodology has been applied previously to define the environment of various communities and explore ways that the local community can support intervention efforts in HIV/AIDS studies in South Africa [23, 26, 32, 33].

### Study setting

This study was conducted in Jamestown and Usshertown (Ga Mashie) in Accra, Ghana. This study is part of a large mixed-methods study aimed at improving the contextual understanding of diabetes and related risk factors in Accra [34]. Jamestown and Usshertown are indigenous ethnic Ga communities with multiple generations of families living together in large households [10, 35]. Educational attainment is generally low, and fertility is high [10]. The dominant occupation among males is fishing, while females generally engage in petty trade [36–38]. Jamestown and Usshertown are densely populated, with over 120,000 people occupying one square mile area [34].

### Data collection

#### Cognitive mapping

Eligible respondents were those individuals who had lived in the community for at least ten years and were 18 years or older at the time of data collection. Respondents must have lived in the community long enough to develop environmental awareness and a deep connection with their space. A total of 43 participants were recruited for both the CM-FGDs and the regular FGDs. The groups were conducted primarily in Ga, the dominant local language. Participants were selected according to diverse characteristics including age, gender, locality, place of birth, marital status and occupation. This participant selection was based on the known environmental risk differentials associated with demographic characteristics [39]. Typically, physical activity, alcohol consumption and other risk factors are influenced by gender, age, occupation, locality and history [25].

Participants were initially recruited and trained as a group for one hour by the research team. The CM-FGD process was explained to them during this time. Participants were asked to independently draw any aspect of their daily environment that reflected the content of their own environmental space. Community members were not expected to draw the maps to scale. They were only expected to express their views through drawing. Participants were informed that the drawings did not have to be perfect or comparable. The drawings were individual expressions of their perspective. Researchers allowed participants ample time to complete their drawings – each group took between three and seven days to complete their drawings. Another date was scheduled after the training, usually between five and ten days later, for the group discussion. This allowed study participants ample opportunity to reflect on their environments and express their perspectives on paper. Researchers considered each individual drawing on its own merit.

During the CM-FGDs, researchers displayed all CMs created by the participants on a board. Each participant was then invited to explain their drawing(s). Other participants raised questions that generated conversation about the environment, with various themes emerging. In addition, a semi-structured interview guide was developed to facilitate the CM-FGDs. Participants were invited to talk about thematic areas raised in their drawings, including environment, community, food and the risk of diseases. With the help of a few probes, the conversations touched on most of these thematic areas. The FGDs lasted between 60 and 130 minutes.

The drawings were photographed with the participants’ permission, and the discussions were recorded. Each CM-FGD involved four to eight participants. All CM-FGDs were conducted in November and December 2022 in the Ga-Mashie Development Agency (GAMADA) office, a community-based organisation and a safe space where maps could be displayed.

#### Food trader FGDs

Upon initial analysis of the CM-FGDs, we identified several references to food environments and felt it necessary to incorporate the perspectives of food traders who operated in the community. Therefore, we conducted four additional FGDs with market women (female), food vendors (mixed group) and butchers (men) to triangulate multiple perspectives on the food environment. Participants for the FGDs were recruited with the support and guidance of the ‘market queen’, the traditional leader of the London market in Jamestown. An interview guide was developed for the CMFGDs and FGDs. The interview guide has been attached as supplementary material.

All CMs drawn by the participants were carefully labelled and kept securely as data. All FDGs were audio-recorded with electronic devices, transcribed and translated from Ga to English. The content of the CM-FGDs and the additional food trader FGDs was analysed thematically. Materials for the study included transcripts, detailed field notes and reports from debriefing sessions after each day of fieldwork. The analysis was conducted by a team of public health professionals and a population scientist with GIS and qualitative methodology expertise. The analysis was undertaken using ATLAS—ti version 7.

#### GIS

The research team used GIS to document the physical environment objectively and allow comparison with the participants’ drawings. The objective maps were created using Quantum Geographic Information Systems (QGIS) software.

### Ethical approval

Ethical approval for this research was granted by University College London (UCL) (21541/001), the Noguchi Memorial Institute for Medical Research (NMIMR) (21541/001) and the Ghana Health Service (GHS-ERC 017/02/22). All participants were asked to provide written informed consent; those who could not sign the consent forms were presented with ink to thumbprint their consent. Participants were also told they could opt out of the study at any time and would not be disadvantaged. To provide privacy to our study participants, interviews were conducted in the safe premises of GAMADA. GADAMA is located a few metres from both communities. GAMADA staff allocated one facility to host all interviews. Audio files and transcripts were secured on password-protected computers. We assured our participants that all conversations were safe and secure. The interview guide for the CMFGDs and the FGDs has been added as supplementary material.

### Data processing and analysis

The first phase of coding involved reading and identifying ideas within each transcript. The second phase involved constant comparison of the codes across transcripts [40]. This study applied two types of coding: first, to identify codes or ideas already present in the literature [3, 9, 41, 42], which were denoted as deductive codes; second, to identify ideas unique to this study and context, which were labelled as inductive codes.

In the third phase of analysis, we grouped similar ideas. This allowed researchers to understand the complexity and breadth of ideas about a particular thematic area. Blocks of ideas expressed in a specific area formed the organising themes. The main themes clustered around defining the community, the nature of the built environment, and its effects on health [27]. Analysis of the CM-FGDs and FGDs was combined.

## Results

### Characteristics of participants

A total of 43 individuals participated in the FDGs – 25 women and 18 men. Of these, 32 had been born and raised in Jamestown/Usshertown, and the other 11 participants had been born outside these two communities but had lived in the study communities for more than 20 years. Most participants (*n* = 17) were < 35 years of age at the time of data collection, and ten were between the ages of 56 and 65 years. A high number of participants (*n* = 16) had attained secondary education. The remainder had attained primary education. Only a small number (*n* = 4) had attained tertiary education. Only three people worked in the formal sector; three participants were students, and the rest were employed in the informal sector, mainly as traders and fisherfolk. See Appendix 1 for full demographic details for all participants.

**Fig. 1:**
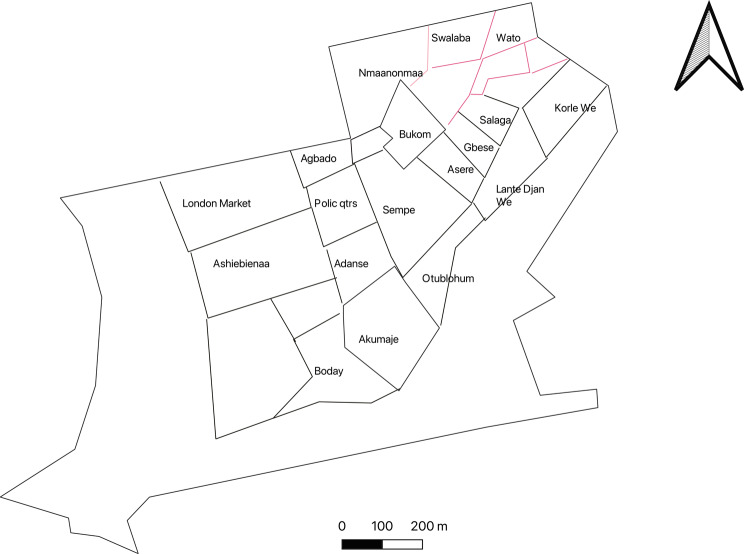
Community neighbourhoods in Jamestown and Usshertown. Source: Care Diabetes map, 2023

**Fig. 2:**
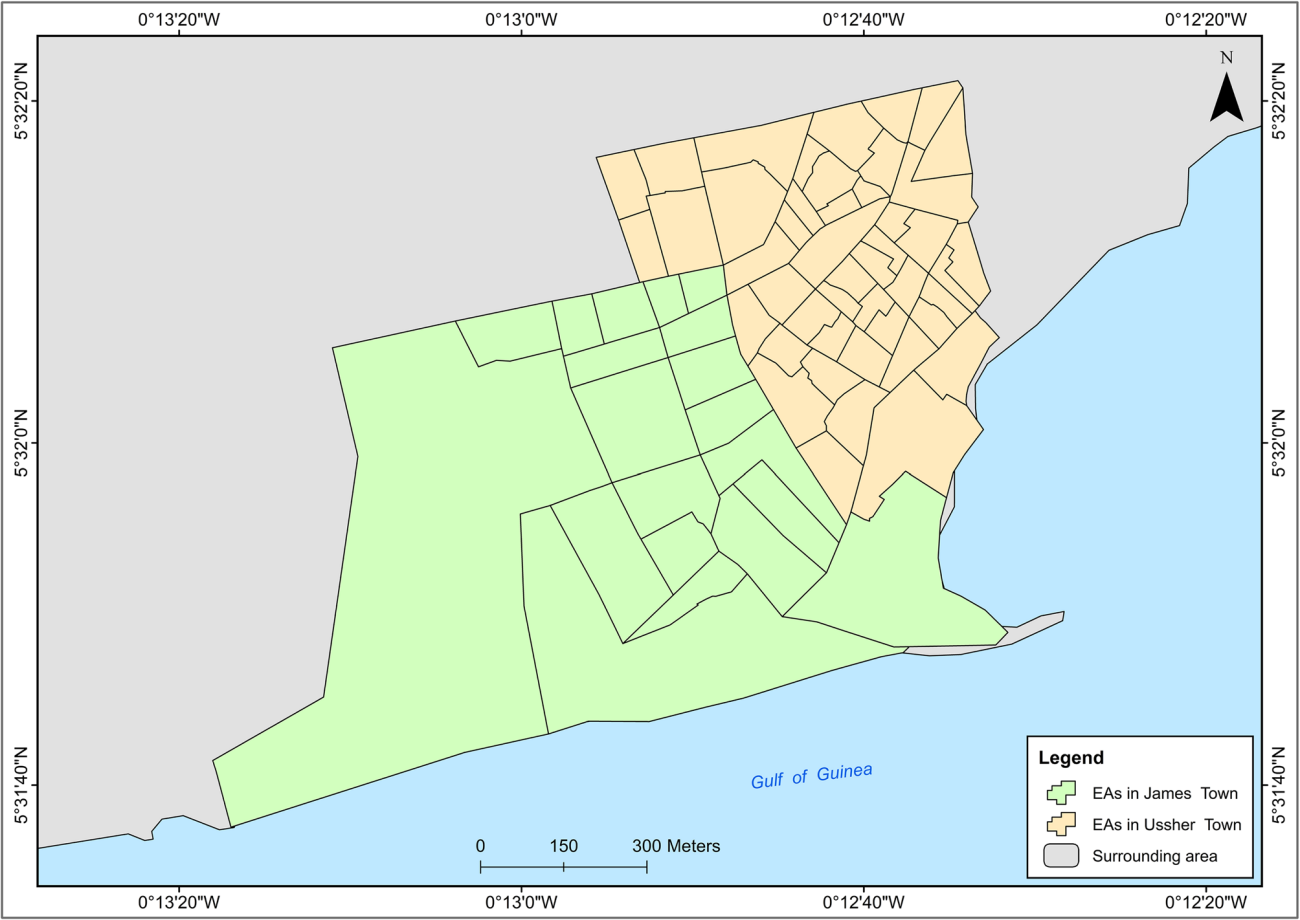
Administrative demarcation of Jamestown and Usshertown

**Fig. 3:**
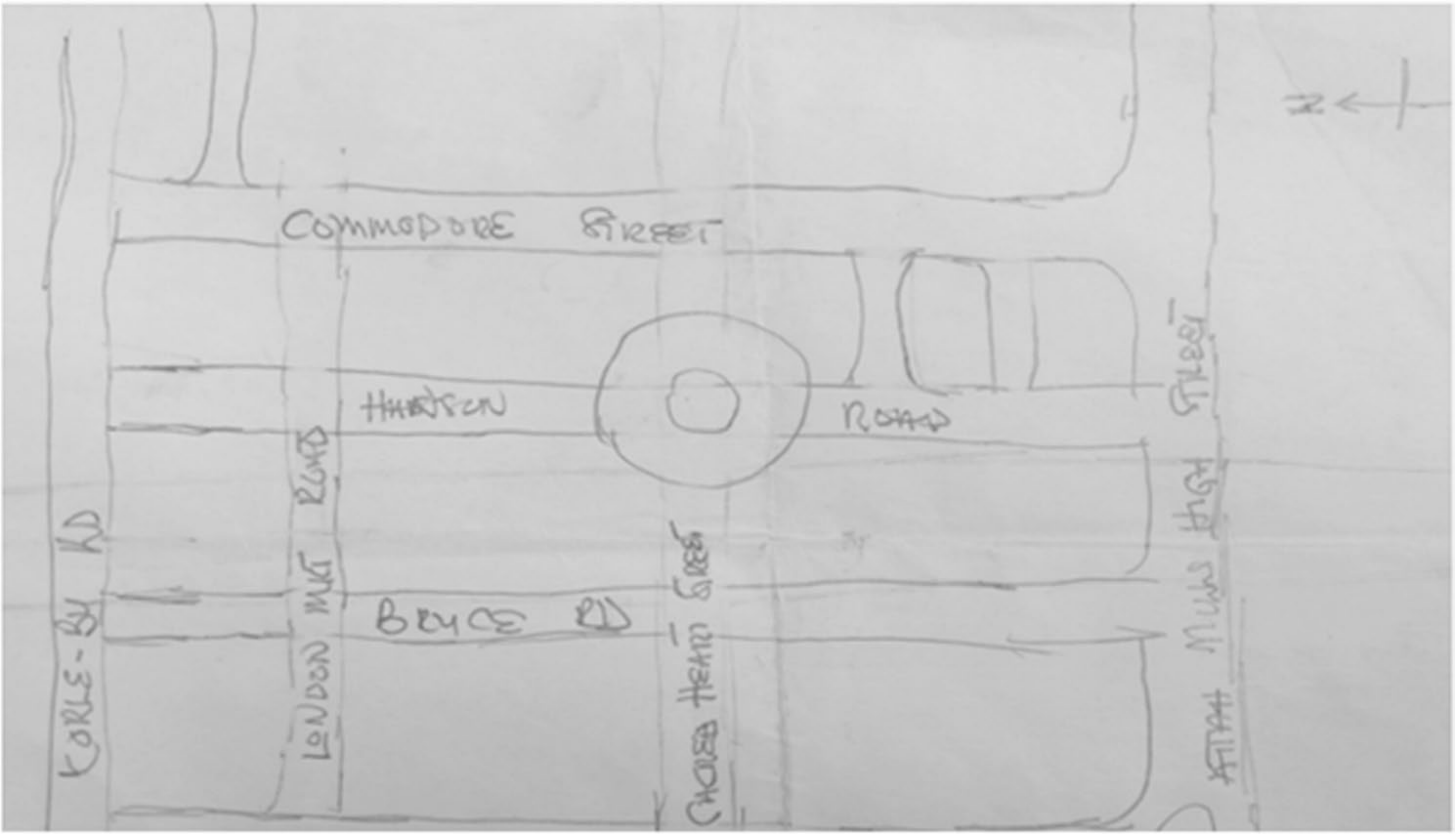
Sketch depicting connectivity in Jamestown. Map produced by woman from Jamestown

**Fig. 4:**
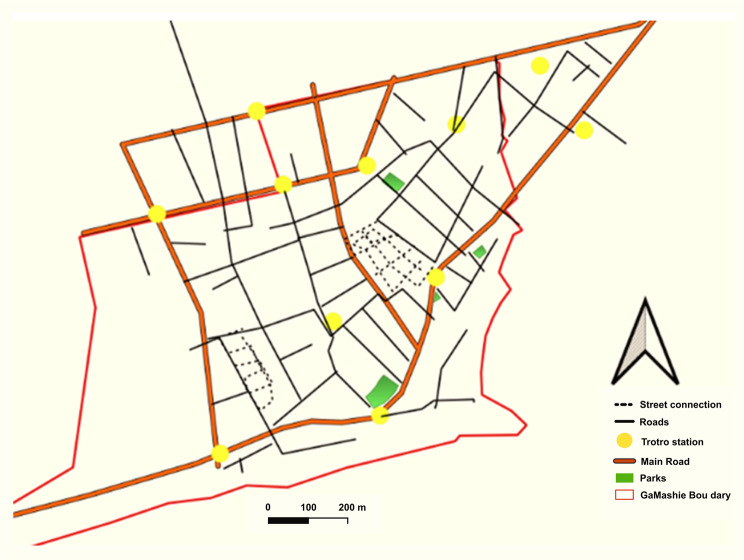
Interconnectivity maps generated with GIS by the research team

**Fig. 5:**
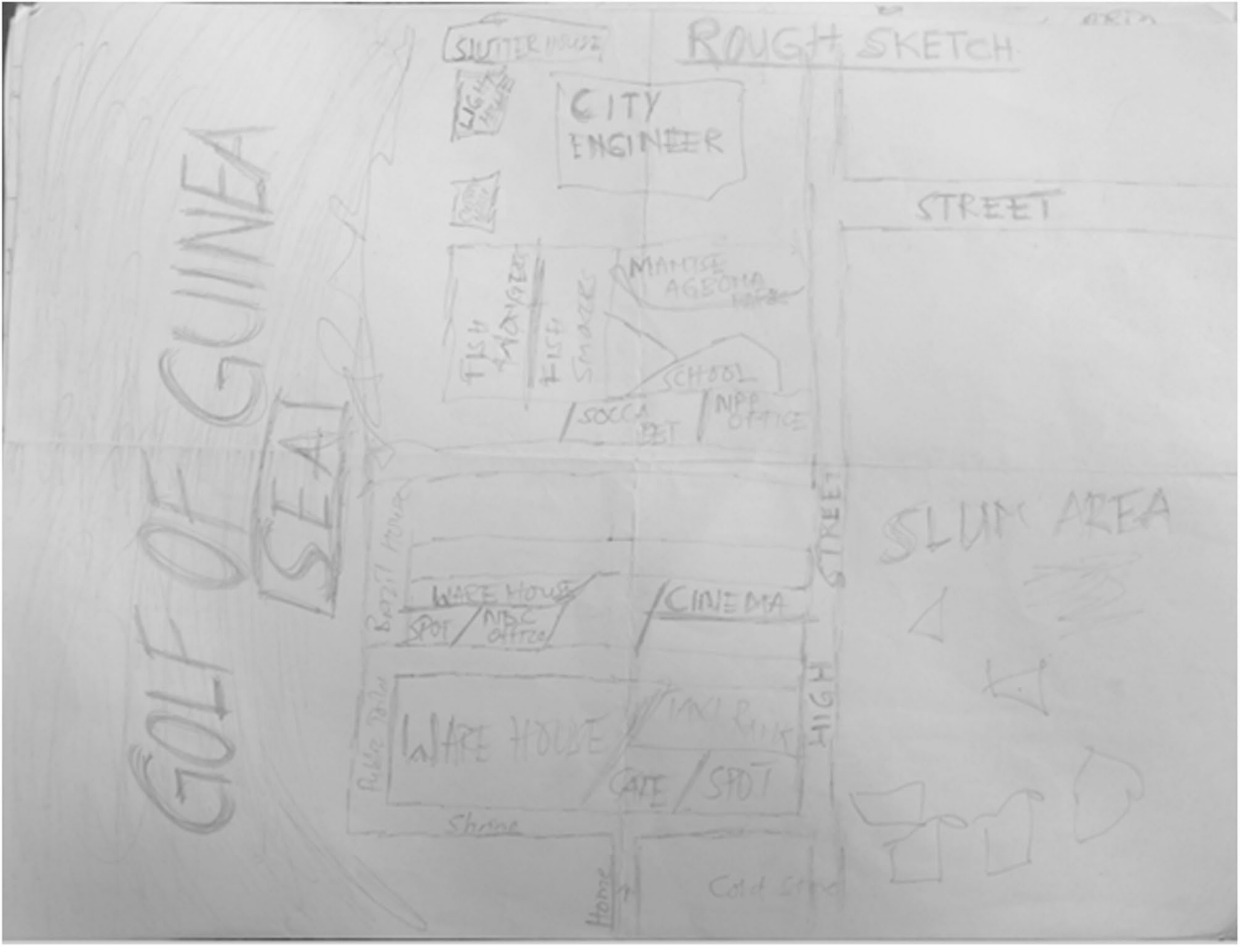
Cognitive map showing social spaces in the study communities

**Fig. 6:**
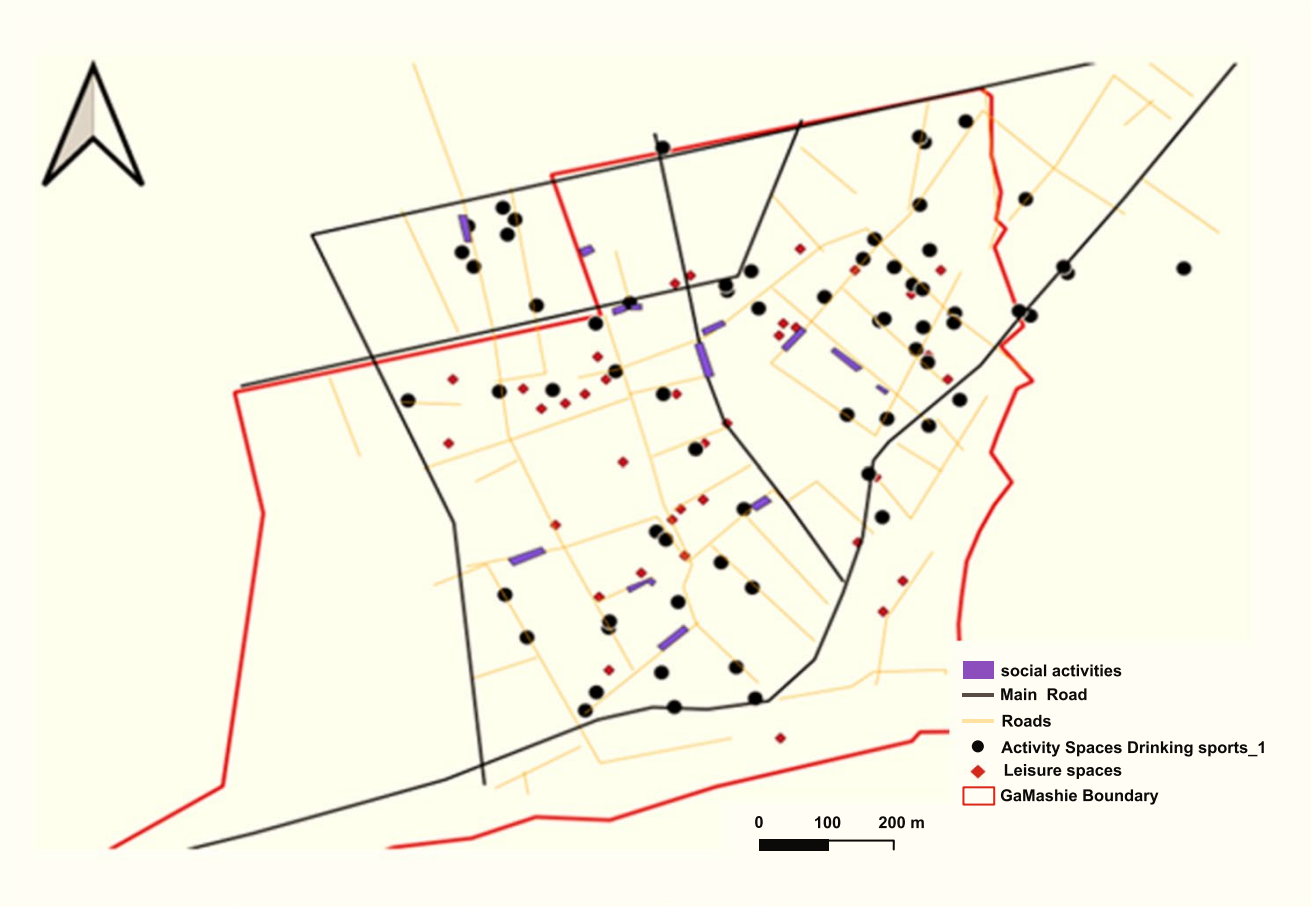
GIS-generated map showing social spaces in the community. Source: Care diabetes project, A social map of Jamestown and Usshertown

Several thematic areas dominated both the CM-FGDs and the trader FGDs. Key areas included lay perspectives of the community, descriptions of the built environment, environmental stressors, the food environment, and the perceived risk of diabetes and CVDs. A comprehensive thematic table showing all the themes and codes is attached as supplementary material. Figures 1, 2, 4 and 6 accurately represent the environment generated using QGIS to provide context to the qualitative discussions. Figures 3 and 5 are sample CMs produced freehand by community members.

### Perceptions of the definition of a community

Participants reported that a community is based on the built environment, ethnicity/clans, history and social behaviour. They emphasised that individuals must share community traits such as language and food to be seen as a community.

“A community is a group of people who may be many or not depending on the piece of land they have. They may be 1,000 or less than 1,000; it is a community. You can identify them with characteristics like their language, food and way of doing things. With my community, for example, you can identify us with our way of life, the way we dress, the food we eat among others.” Woman from Jamestown, FGD 4.

“As far as you can be found in one geographical area, and say that you recognize these people with these traits, it is definitely a community.” Man from Usshertown, FGD 4.

Participants noted that the two study communities (Jamestown and Usshertown) comprise people of the Ga ethnic group with different historical experiences. Participants referred to Jamestown as *Ngleshie*, British Accra; and Usshertown as Dutch Accra. They further subdivided Jamestown and Usshertown into clan units, clustered in specific locations of both communities. Sometimes, the community was defined in terms of these subdivisions. Participants mentioned the Akumaje, Sempe, Gbese, Otublohum, Asere and Abola clans. Sometimes, certain behaviours and practices were associated with these clan units.

“Where you [addressing another participant] live, we call that whole area, Akumaje.” Woman from Jamestown, FGD 2.

### Descriptions of the built environment of Ga Mashie

Participants described the built environment according to neighbourhoods, connectivity, population density and the food environment. They deployed paths, edges, districts and nodes to explore the characteristics of the community.

#### Neighbourhoods in Ga Mashie

Participants used edges to indicate the boundaries of the neighbourhoods. Paths were used to show the street and road networks in the community. Nodes were used to show bus stops. Finally, boundaries were used to identify the geographic boundaries of the clan units within both communities. Figure 1 is an approximation of the neighbourhoods that was generated by the research team. During the daily debriefing sessions, the team developed a map to show the neighbourhoods that had been mentioned by community members. The community map in Fig. 1 differs substantially from the official map of Jamestown and Usshertown in Fig. 2. Figure 1 has 29 areas, while Fig. 2 has 82. Also, the Enumeration Area (EA) boundaries in Fig. 2 have no real meaning to the community. Community members did not use any known landmark to define community.

#### Connectivity or paths in Ga Mashie

Figure 3 is a sample map showing a participant’s perceived connectivity of Jamestown and Usshertown. Figure 4 is the GIS-generated map. While Figs. 3 and 4 look aesthetically different, they convey the same message that the community is interconnected to a high degree. The maps display the main roads, community vehicular routes and footpaths. There are 11 bus (*trotro*) stations in the communities. The dominant vehicles in the community are commercial motor bicycles, tricycles and minivans. Most Jamestown and Usshertown alleys are paved. This makes the roads and the interconnections accessible at any time, under any weather conditions. Participants reported that, before the pavements were laid, the community experienced flooding whenever it rained.

#### Population density in Ga Mashie

The communities were described as overcrowded due to the density of people seen on the streets, in houses and in sleeping rooms. The residential blocks are large structures consisting of several single rooms. These structures are usually family houses that are shared by extended families across three to five generations. A household (on average, three members) often inhabits a single room and sometimes shares the space with an extended family member. The quotes illustrate these characteristics.

“Our household is in a family house. I have been given [one] room that I stay in, but because my sister has not been given hers, I share it with her and also with her children.” Woman from Usshertown, FGD 4.

“I live in [one] room with my husband, and we have four children who are not married; we all live there and that increases our population.” Woman from Jamestown, FGD 5.

#### Social spaces of Ga Mashie

Social spaces refer to places or centres where people gather and interact. Participants mentioned spaces such as pubs (‘drinking spots’), cinemas and ‘parliaments’ (Figures 5 and 6). They also mentioned social events such as funerals and naming ceremonies. These temporary spaces could be set up anywhere in the community, including in streets, courtyards and car parks.

Figure 5 depicts a CM drawn by a respondent that shows two drinking spots, a café, a soccer betting shop, a warehouse, political party offices and other spaces. The only social space that appears twice on the same map is the drinking spot, emphasising its abundance in the community. The map also indicates the offices of the dominant political parties in Ghana (i.e., the New Patriotic Party (NPP) and National Demographic Congress (NDC)). These are sheds that are funded by the two parties where people sit and mingle. These spaces are often draped in the paraphernalia of political parties. Sometimes, local community members called these spaces ‘parliament’. Participants reported that there was limited activity within these spaces during the day. They came alive in the evenings when people returned from work and were available to socialise. Participants observed that men and male activity dominate these social spaces.

“With this parliament thing we are talking about, we have boys–boys joint, normal community groups joint, then we have the political joint like the NPP, NDC and CPP joints. Looking at the NPP joint, the people who sit there are big men not in the sense of the job they do but they have political influence. Because of the political influence they wield, they just sit there and get some stipends from the political leaders and come and share it. They will be sitting there and political leader will probably come and give them let say 100 cedis [US$8] to share. They go and sit there and maybe there is a political … even that is going to come on, if there is a job opportunity, they will be like ‘oh, we know this person because he has been coming to sit here with us so give it to him’. These are some of the benefits these people get by going to sit there.” Woman from Usshertown, FGD 5

The map depicted in Figure 6 is saturated with community leisure spaces and drinking spots. The study mapped 37 leisure spaces and 67 drinking spots in the two communities. Participants reported that almost all funerals occurred on community vehicular roads, which limits mobility.

“As for weddings, you hear of it once in a while. Funerals happen often and during funerals we drink and eat.” Woman from Usshertown, FGD 4

“Yes, after the rituals, you see, we have some two to three hours to have fun. That is where you see people smoking, mixing [alcoholic cocktails. That happens on the first day of the funeral. On the first [day] of the funeral, there is a lot of drinking and eating.” Man from Usshertown, FGD 4

### Environmental stressors of the built environment of Ga Mashie

Participants described two significant environmental hazards due to overcrowding: hazardous heat and sound.

#### Hazardous community heat

Participants expressed strong opinions concerning heat intensity in the communities and how that affected their health. Heat, together with environmental stressors, was mentioned 35 times in the FGDs. Participants attributed heat in the community to population density, the built environment and related human activities, with population density cited most frequently (*n*=8). Other causes of heat included the spatial organisation of the neighbourhood, domestic and industrial cooking, the presence of a slaughterhouse, climate change and the weather. The effects of heat in the community included sleeplessness, mental health conditions, skin rashes and sleeping outside. Participants reported that heat drove people outdoors, and that heat from room overcrowding kept community members outdoors most of the day.

“Your aunt could light the coal pot to cook something. She cannot place it in the sun, so she will place it in front of you so we can both sit there. The fresh air cannot come through because the fire is in the way, so we are all experiencing the heat.” Woman from Usshertown, FGD 1

“From here to the house, there is a lot of heat and when you get home, the house is hot. Where will you go to?” Woman from Jamestown, FGD 2

#### Hazardous community noise

Hazardous noise was mentioned most frequently by participants as an environmental stressor. Overall, hazardous noise was associated with 83 different themes: primarily behavioural factors such as shouting, religious activities, industrial activities; vehicular activities, social activities such as funerals and naming ceremonies, sporting activities, alcohol vendors who play loud music all the time, and even family fights. Participants raised concerns about excessive and unregulated noise pollution at all times that pose a potential health and safety risk. Some participants shared their sense of powerlessness in response to the high volume and intensity of noise within the neighbourhood.

“As for the churches, I wouldn't want to mention them because they will say I don’t like God. Regarding the churches, all-night programmes used to be only Fridays but now they do it Wednesdays and Fridays. So just imagine if you live closer to a church. We are becoming used to it because we have lived with it for quite some time. Also, they are playing their stuffs it vibrates a lot. As for the funeral, there are those who close theirs early but others will not. So how can you sleep on Sunday and go to work on Monday if you work? Some of them intentionally do the funeral for Lengthy hours to show off. But when I was growing up, it was not like that. Funerals used to close at around 5pm, and during the wake that music is played in the evening. But now there has been a lot of changes in that aspect.” Man from Usshertown, FGD 6

#### Noise, poor sleep, headaches, increased blood pressure

Participants believed that noise negatively affected several aspects of the community. They associated hazardous noise with a lack of peace of mind, leading to personal discomfort and psychological distress. This theme was recorded seven times in the FGDs and was only mentioned in reference to noise. Study participants linked noise intensity to headaches and a host of CVDs, including hypertension, chest pains and heart attacks. The quote below shows how community members link mental health stressors to heart health.

“When I sit down and you are standing close to me, making noise, I feel like I have a headache. It feels like you are exerting some pressure. It is like in my mind, there is a lot of noise, and I feel disturbed.” Woman from Usshertown, FGD 2

“It also brings about a headache, and sometimes you become startled as a result of the noise, and that can result in a heart attack. When someone shouts so loud, there can even be a rise in your blood pressure, which can create problems.” Woman from Jamestown, FGD 6

### The street food environment in Ga Mashie

Food is a major aspect of community life. Participants described food availability, consumption time and taste as the main drivers of the changes in food culture. Participants noted that strong tastes drove the food culture, influencing food preparation. They noted the increased use of processed condiments and artificial flavouring (e.g. A1, Maggi), which were foreign to traditional food preparation methods. According to community members, using these condiments has shifted demand towards “tasty food” that has a “delicious taste”, which they associated with lifestyle diseases like diabetes. Street food vendors constituted the main food outlets in the community, selling ready-to-eat meals such as jollof rice, noodles, bread and kenkey (a popular fermented maize mixture with dough moulded into balls in a corn husk). Community members discussed the changes in the food culture, specifically the emergence and dominance of fried foods.

“Maggi, A1 and different spices. They use it to prepare the food just to make it taste good, but it is not good for our health.” Food vendor from Jamestown, FGD 8

#### Increased consumption of artificial seasoning

Participants reported that competition among street food vendors influenced the use of harmful cooking practices. Food vendors, however, argued that they only cooked according to the tastes of community members. Food vendors suggested that cooking practices adopted for commercial purposes differ from those used for domestic purposes. This highlights awareness of the risk associated with using strong, artificial flavours.

Many community members mentioned preferences for street foods as a notable risk factor for the rise in CVDs.

“Also, we use Maggi in almost all the food we prepare recently, which wasn’t like that in the olden days.” Food vendor from Jamestown market, FGD 7

“If the food isn’t tasty, they won’t buy, so the onus lies on you to make it tasty. Hence, Maggi must be used to get the desired taste.”Food vendor from Jamestown, FGD 8

#### Increased consumption of out-of-home meals

The high population density and lack of cooking units were cited as key factors associated with purchasing and consuming meals prepared outside of the home (‘out-of-home’ meals). Participants explained that there was some family pressure to share home-cooked food. Because living spaces are often small, overcrowded, and poorly ventilated, cooking typically occurs in courtyards. In these shared spaces, other family members may join in, and participants are often expected to share meals with their extended relatives. These conditions were viewed as obstacles to home cooking, making it costly and inconvenient, leading to greater reliance on food prepared outside the home.

“Currently, things are expensive in the world, and also people don’t get enough money for upkeep; cooking has become quite difficult. So people intentionally play with your kids so that you give them some after cooking, that’s when they realise that you are cooking.” Food vendor from Usshertown, FGD 8

#### Late-night meals

The risks related to food were due not only to cooking practices and taste, but also to the time that food is consumed. Participants noted that the communities were very vibrant at night and many social events continued late into the night. They saw eating late as a community behaviour associated with the risk of diabetes.

“When I eat late, I suffer from heartburn, and I also learnt that eating late raises one's sugar level, which leads to diabetes. Also, you don’t have enough rest.” Man from Jamestown, FGD 6

#### Low physical activity

Low physical activity and limited opportunities for physical activity were cited frequently in the CM-FGDs. Participants observed that the communities’ parks, gyms and fitness centres only served younger male adults. There were generally not enough opportunities for women and older people to undertake physical activities. Even the pavements were occupied with street food, limiting walking and movement.

“Yes. At the roundabout there is space. They call the place Buduemu. You can exercise there but you need to pay for the place before you get to [exercise]. But at the roundabout, you will see only boys.” Woman from Usshertown, FGD1

Diabetes was mentioned eight times, with all occurrences associated with the social environment and lifestyle choices.

“I think we have hypertension resulting from too much fat, and then diabetes as a result of the sugary stuff like drinks.” Woman from Jamestown, FGD 2

“It’s like *kenkey* contains sugar, so when you over eat it, you may end up with diabetes.” Food vendor, Usshertown FGD 8

“Yes, when you realise what the customer wants, you do it for them. So if you go to the market and buy something else, you destroy your own market.” Food vendor from Jamestown FGD 8

“The food vendors prepare the food with a profit-making mindset. So they use much oil, Maggi and beef, but she prefers dried fish. So the food vendors use the beef and all those ingredients to make the food attractive and tasty. But that is what makes us sick but we have no idea.” Man from Jamestown, FGD5

“I think there is a shift in taste.” Man from Usshertown, FGD 4

### Discussion

Communities thrive in spaces where people feel safe and valued. In turn, environmental harmony creates the safety and security needed to maintain optimal health [43]. Unfortunately, overcrowding and unregulated use of space generate stress and increase the risk of lifestyle diseases [43]. This is amplified by poorly designed cities [38, 44, 45] – which create environments where excessive noise, traffic, fear of crime and poor air quality represent risk factors for obesity and other CVDs [46].

This study documents community perspectives on the effects of a hostile environment on health in urban Accra. Community members believed that the physical environment and social factors contribute to the development of diabetes and CVDs. These risk factors were identified at the individual, household and community levels.

Risk factors are woven into community behaviour patterns through numerous platforms and community engagements. Notable diabetes and CVD risk factors, such as alcohol consumption, form an integral part of community life, for example at funerals. While acknowledging the history and culture of the community, participants also noted changing dietary patterns and lifestyles that increase the risk of diabetes and CVDs. Conversations predominantly focused on how changing food practices and behaviours contribute to risk factors associated with diet.

The community's complex perspectives on the built environment highlight the challenges in scientific conceptualisation, measurement and understanding of the built environment and related risk factors. This may partly explain why a simple physical count of liquor outlets, parks, walkways and other environmental measures may not always directly predict the influence of the environment on CVD risks [47].

Previous studies have established the relationship between the built social environment and the risk of diabetes [48]. Ecological models emphasise the influence on behaviour at the level of the individual, group, community, built environment and social environment [9]. Many studies show that physical inactivity and the food environment are key risk factors for diabetes [48]. The built environment influences the risk and management of diabetes through the creation and availability of walkways, gyms and parks, and the concentration of food outlets. For example, the concentration of fast-food outlets in the UK is significantly associated with diabetes risk [49]. Within the social environment, hazardous noise is known to elevate stress levels and sleeplessness, and this has been associated with the risk of some CVDs and diabetes [50]. Participants made these associations to explain the risk of diabetes and CVD within urban Accra. These local understandings of the built environment provide an opportunity to engage community members and prescribe meaningful solutions.

Community heat and noise are noteworthy themes under which participants expressed powerlessness: people felt the impact of these environmental characteristics was beyond their control. The built environment and intense human activity produced a challenging environment in the study communities. People lived in multigenerational households, sometimes owned by whole clan units. No single individual necessarily had control over what occurred in these dwellings. However, the shared spaces offered some advantages too, such as giving people somewhere to reside for free in increasingly expensive urban Accra. The disadvantage is that community members do not choose the people they must live with, and they must do so indefinitely. Sometimes, several adult siblings shared a single room with their children because they inherited that space from a parent and therefore had an equal stake. In several other studies, powerlessness is associated with difficult inner-city neighbourhoods and is attributed to crime, violence and instability [51, 52]. In this study, however, participants associated powerlessness with the regular functioning of their community regarding noise, heat and even food. Participants’ inability to control the number of food outlets in their community and the processed goods available resulted in a harmful food environment that increased their risks of diabetes and other CVDs.

Lack of autonomy over their dwellings and other spaces within their community fuelled the perspective that elements within the environment cannot be controlled, even if the community had created those spaces. However, participants cited several examples where they could assert some control over community-level problems. For example, once every year, the community bans drumming and noise-making for a whole month preceding the annual festival. These bans are upheld with religious verve. Similarly, to control the noise level in neighbouring areas, some communities permit funerals only once per month rather than occurring at any time. This same energy could be channelled into managing other community-level phenomena that affect the health of large groups.

While there may be differences in context, it is noteworthy that community-level strategies have been proven to work in communities not far from Jamestown and Usshertown. Several active community organisations, such as market women’s groups, fishermen’s groups, youth groups and others, could be sensitised to advocate for community-level reforms through the traditional council and the Accra Metropolitan Assembly (AMA). Indeed, GAMADA exists for such a purpose, and it has successfully led several community-level initiatives.

### Strengths and limitations

Using multiple methods allows one to observe the environment from various perspectives; however, synthesising observations from multiple perspectives can be challenging. As the study communities are multicultural, different language competencies were required to communicate effectively with participants. It was initially quite challenging to ask older community members with limited formal education to draw, but they eventually enjoyed the process.

While findings from this research may have implications for similar urban poor contexts in Ghana and beyond, the study was not intended to generalise the findings. The aim was to understand nuanced community perspectives on how the environment may influence the risks for diabetes and CVDs.

## Conclusion

This study highlights the vital role of environmental factors in shaping people’s health behaviours. Although people understand these factors, they feel powerless to change or control them. Articulating the problems is a necessary first step towards identifying solutions, such as limiting noisy and late-night events or actively promoting healthier foods at fast-food outlets.

## Supplementary Information

Supplementary Material 1.

## Data Availability

Please data will be made available upon requests. Prof. Edward Fottrel is the Principal investigator of this project and should be contacted for access to this data. e.fottrell@ucl.ac.uk.

## Appendix 1

**Table 1:** Characteristics of study participants

Participant ID	Gender	Age	Marital status	Place of birth	Occupation	Religion
FGD 1.1	Female	68	Single	Jamestown	Trader	Christian
FGD 1.2	Female	69	Single	Jamestown	Trader	Christian
FGD 1.3	Female	63	Widowed	Jamestown	Kenkey seller	Christian
FGD 1.4	Female	57	Married	Jamestown	Trader	Christian
FGD 1.5	Female	54	Married	Jamestown	Trader	Christian
FGD 2.1	Female	51	Widowed	Jamestown	Trader	Christian
FGD 2.2	Female	69	Married	Jamestown	Trader	Christian
FGD 2.3	Female	63	Married	Jamestown	Trader	Christian
FGD 2.4	Female	57	Married	Jamestown	Trader	Christian
FGD 3.1	Male	58	Married	Usshertown	Fisherman	Traditionalist
FGD 3.2	Male	60	Widower	Usshertown	Fisherman	Traditionalist
FGD 3.3	Male	62	Married	Usshertown	Fisherman	Christian
FGD 3.4	Male	71	Married	Usshertown	Fisherman	Traditionalist
FGD 4.1	Male	20	Single	Jamestown	Student	Christian
FGD 4.2	Male	21	Single	Jamestown	Student	Christian
FGD 4.3	Male	24	Single	Usshertown	Fisherman	Christian
FGD 4.4	Female	24	Single	Jamestown	Trader	Christian
FGD 4.5	Female	26	Single	Jamestown	Seamstress	Christian
FGD 4.6	Male	53	Married	Jamestown	Mechanic	Christian
FGD 5.1	Male	46	Single	Usshertown	Navy	Christian
FGD 5.2	Male	38	Single	Usshertown	Printing press	Christian
FGD 5.3	Female	35	Single	Usshertown	Trader	Christian
FGD 5.4	Female	31	Married	Cape Coast	Trader	Christian
FGD 5.5	Female	40	Single	Usshertown	Trader	Christian
FGD 5.6	Male	18	Single	Usshertown	Student	Christian
FGD 6.1	Female	25	Married	Jamestown	Trader	Christian
FGD 6.2	Female	31	Married	Labadi	Food vendor	Christian
FGD 6.3	Male	35	Married	Usshertown	Teacher	Christian
FGD 6.4	Male	27	Single	Teshie	Mechanic	Christian
FGD 6.5	Male	24	Single	Jamestown	Student	Christian
FGD 7.1	Female	37	Single	Jamestown	Trader	Christian
FGD 7.2	Female	68	Married	Jamestown	Trader	Christian
FGD 7.3	Male	60	Married	Jamestown	Trader	Christian
FGD 7.4	Female	64	Widowed	Jamestown	Trader	Christian
FGD 8.1	Female	49	Married	Jamestown	Kenkey Seller	Muslim
FGD 8.2	Female	32	Single	Jamestown	Tea Seller	Christian
FGD 8.3	Female	54	Single	Jamestown	Waakye Seller	Christian
FGD 8.4	Female	48	Single	Jamestown	Jollof Seller	Muslim
FGD 8.5	Female	27	Married	Jamestown	Beans Seller	Christian
FGD 9.1	Male	37	Married	Jamestown	Butcher	Muslim
FGD 9.2	Male	28	Single	Nungua	Butcher	Christian
FGD 9.3	Male	32	Single	Ashaiman	Butcher	Christian
FGD 9.4	Male	58	Widower	Usshertown	Butcher	Christian

## Abbreviations

CM-FGDs: Cognitive Map Focus Group Discussions
FGDs: Focus Group Discussions
GIS: Geographic Information Systems
CVD: Cardiovascular Diseases
GAMADA: Ga-Mashie Development Agency
UCL: University College London
NMIMR: Noguchi Memorial Institute for Medical Research
NDC: National Democratic Congress
NPP: New Patriotic Party
