# Supplementary material for: Community perspectives on the built environment, community stress, and the risk of diabetes and cardiovascular diseases in Accra, Ghana
Source: BMC Public Health. 2025 Oct 14;25:3469. doi: 10.1186/s12889-025-24106-z (PMC12522272; doi:10.1186/s12889-025-24106-z)
Supplement: Supplementary file 1 — Supplementary Material 1. [file 12889_2025_24106_MOESM1_ESM.docx]

|  | Objective | Methods | Activities |
| --- | --- | --- | --- |
| 1 | Objective Community mapping | GIS/structured observation | 1. Mapping recreational areas 2. Mapping physical activity spaces 3. Designated community sitting areas. 4. Areas designated for social events, markets, 5. Mapping out children’s playground etc 6. Mapping out venues for community social activities 7. Mapping community street walkability |
| 2. | Subjective Community Mapping | FGDs | 1. Understanding the community perspectives on the use of space: connectivity, net residential density, land-use mix,   street accessibility, and provision of sidewalks.   1. Understanding norms around physical activity, the food env 2. Developing a community map based on 3. Community reported criminal activity 4. Night time community activity 5. Talking about community alcohol behaviour |
| 3. | History of community Interventions | IDI | Conducting interviews with key community stakeholders |
| 4. | Photovoice/Cognitive mapping |  | FGD participants |
|  | 5. The food environment | GIS mapping | 1. Mapping all foods, cooked foods, uncooked foods, drinks, grocery shops, |
|  | 6. Alcohol environment | GIS Mapping | 1. Mapping the sales point of alcohol 2. Mapping Advertisement of alcohol |
|  |  |  |  |

Environment and Context (Leads-Kushitor and Yacobi)

Map the study area, including healthcare facilities and the digital landscape.

**Objective One**

GIS Mapping intensive community mapping

**Objective Two**

*Community Recreation*

1. What are the common recreational activities in this community
2. Where care the places of recreation in the community
3. Can you talk about the time of the day, week, year that people engage in recreation mostly.
4. Are there spaces designated for sitting
5. What typically happens in community sitting spaces

*Social events*

1. Can you describe the organisation of social events in this community,
2. Which events are the most important
3. How frequent do people attend these social engagements
4. Can you describe dietary patterns in these social events
5. Can you describe drinking patterns in these events

*The Food environment*

1. Can you describe the food environment in this community
2. What are dietary consumption patterns
3. How do people access food in this community
4. Would you consider the

*Community physical activity space*

1. Let us talk about physical activity
2. What are your perspectives on physical activity engagement in the community
3. What are the commonest physical activities
4. Where do people normally carry out their physical activities in the community, are there designated spaces for physical engagement
5. Do you feel the community has ample opportunities for people to engage in physical activity
6. Is there a community desire to engage in physical activity
7. How would you describe the walking experience in the community, is there ample space to walk, are there designated spaces to walk around the community etc

*spatial organisation community*

1. Can you comment on residential arrangement in this community/ housing arrangement, room density, spacing around dwelling etc
2. Can you discuss population density in this community
3. Can you discuss how population density affects the use of space
4. Can you discuss how easy it is to walk in the community from one end to the other.
5. Do you feel that the population density affects our health
6. Can you comment on the level of noise in the community
7. Does noise affect health in this community? Can you identify the areas with the highest noise intensity in the community?
8. How about the heat in the community?

*Community safety and healthy living*

1. Do you feel safe in this community? Probe for the time of day
2. Do you feel safe walking around for physical activity
3. Do you sometimes feel that some could harm you while taking a walk around?
4. Has someone ever broken into your home

**Objective three**

1. Do you remember any interventions in these two communities?
2. What kinds of intervention were carried out?
3. Can you mention specific numbers?
4. What were the nature of the interventions? Methodology
5. Can you name some of the funders?
6. Can you talk about the objectives of the interventions?
7. Do you feel the interventions were complementing each other?
8. Do you remember interventions that were focused on shaping the community/ environment?
9. How many were they?
10. Do you feel these interventions have impacted the community in anyway? How so? /why not?
11. What were your expectations of the interventions

**Objective four**

In 1992, Wang and Mary Ann Burris developed Photovoice based on a combination of Paulo Freire’s notion of “critical consciousness” (a deep understanding of the way the world works and how society, politics, and power relationships affect one’s own situation); feminist theory, which emphasizes the importance of voice; and documentary photography, which is often used to help bring about social change.

1. Critical consciousness about the food environment
2. Reflect, discuss and capture critical environmental influences on food using pictures. The same FGDs I suggest should be used for the cognitive mapping
